# The trouble with IVF and randomised control trials: Professional legitimation narratives on time-lapse imaging and evidence-informed care

**DOI:** 10.1016/j.socscimed.2020.113115

**Published:** 2020-08

**Authors:** Manuela Perrotta, Alina Geampana

**Affiliations:** Department of People and Organisations, School of Business and Management, Queen Mary University of London, United Kingdom

**Keywords:** IVF, Time-lapse imaging, Evidence-based medicine, Add-on treatment, United Kingdom, National Health Service (NHS)

## Abstract

Focusing on the case of time-lapse imaging (TLI), this paper analyses how medical professionals negotiate the use of new ‘add-on’ fertility treatments in light of the limited evidence available. The data produced by TLI technologies is meant to help professionals identify the best embryo to be implanted. Embryo selection is essential in IVF practice for increasing pregnancy rates and reducing the negative effects of repeated failures. More than 5 years after the introduction of TLI in IVF labs, however, there has been no conclusive randomised control trial (RCT) evidence to show that the tools do indeed have a significant impact on pregnancy rates. Nonetheless, many public clinics in the UK have adopted such technologies. Consequently, our research asks: How is the use of TLI tools legitimised by professionals, in light of contradictory evidence? Focusing on 25 semi-structured staff interviews, we argue that professionals use several strategies to legitimise the use of TLI in the clinic without, however, challenging the tenets of evidence-based medicine (EBM) and the value it places on RCTs. Rather, professionals emphasise various advantages that TLI offers, including its use as a lab tool, its potential for knowledge production in embryology, and the role it plays in the management of patient expectations and course of treatment. This paper contributes to debates on the role of EBM in modern medicine and fertility care specifically – an area where this inter-relationship has been underexplored. We conclude by suggesting avenues towards a more nuanced understanding of EBM as it relates to IVF treatment and a rapidly changing biotechnology context.

## Highlights

• Professional legitimation of time-lapse imaging in light of limited evidence.
• Data on professional perspectives on the use of new fertility treatments.
• Article connects IVF add-on debate to critiques of evidence-based medicine.
• In-depth analysis of time-lapse imaging case in UK clinics.
• Randomised control trials still considered gold standard in IVF.

## 1 Introduction

Since the birth of the first IVF baby in 1978, the number of children conceived through assisted reproductive technology worldwide has reached over 6 million. Nevertheless, IVF success rates remain quite low overall. For instance, the UK live birth rate (LBR) was 22% in 2017 (HFEA, 2019a). To increase success rates, countless adjuvant therapies and techniques have been introduced in the last few decades. These are referred to as ‘add-ons’. A recent survey conducted by UK regulator, the Human Fertilisation and Embryology Authority (HFEA), shows that over 70% of patients have had one or more add-ons included in their fertility treatment (HFEA, 2018).

Recently, the efficacy of add-ons has been criticised in the medical literature as lacking robust evidence (Heneghan et al., 2016) and for offering little or poor‐quality information to patients (Spencer et al., 2016). In the UK, subsequent spin-off media responses, including a BBC Panorama documentary (The Fertility Business, 28 November 2016), claimed that the wide use of over 30 add-ons, usually offered at a charge, was aimed solely at increasing clinic profits. The UK fertility sector has been criticised for making ‘false claims of effectiveness to take financial advantage of desperate couples’ (Rutherford, 2017, p. 1850).

Although add-on treatments are widely used in most NHS (public) clinics, debates have focused on commercialisation and additional costs charged by private clinics. Despite treating a mix of privately- and publicly-funded patients, NHS clinics usually do not charge extra for add-on treatments. With limited commercial interests, however, the public fertility sector suffers from a lack of funding, especially in England, where only 35% of IVF cycles are publicly-funded (HFEA, 2019a). Interestingly, criticism of the inclusion of add-ons in state care regimes has been marginal, compared to other treatments lacking decisive efficiency evidence, such as homeopathy, for example (Hansen and Kappel, 2012). Currently, there is little information on the use of add-ons in public clinics, despite the above-mentioned controversy.

To explore how NHS fertility professionals legitimise the use of add-ons in their medical practice, we investigate the case of one of the most diffused add-ons: time-lapse imaging (TLI). TLI tools are laboratory incubators with integrated cameras that take recurrent pictures of embryos during their first days of development. Although TLI has several potential benefits (constant embryo monitoring, quality control, teaching applications), such tools were initially marketed as able to increase pregnancy rates, entering clinical practice with the aim of improving patient outcomes. Nonetheless, two recent Cochrane reviews concluded that there is insufficient evidence of increases in LBRs to justify the use of TLI over conventional incubation (Armstrong et al., 2015, 2019).

The aim of this article is not to take sides in the debate on public funding of TLI. Rather, the intention is to analyse how TLI legitimacy emerges in the IVF professional discourse. Focusing on the analysis of interview data collected in 5 NHS centres in the UK, we will present the strategies that NHS-based professionals use to legitimise their routine use of TLI in their medical practice. We argue in this article that IVF professionals share orthodox views on notions of acceptable evidence, while simultaneously offering alternative perspectives on the role of TLI in improving clinical practice. Their negotiation of legitimacy focuses on three main strategies: presenting TLI as an innovative piece of laboratory equipment; highlighting its crucial role in the production of knowledge; and the ability of these tools to offer additional information to manage patient hopes and expectations.

## 2 The hegemony of the orthodox EBM discourse: a critical overview

The late 20th century saw the diffusion and advancement of evidence-based medicine (EBM) as a response to the lack of standardisation in medical care and concerns that new research was not used in routine clinical decisions. Notwithstanding the hegemonic position of EBM in Western medical discourse, many criticisms have arisen from the medical community itself (see for instance, Feinstein and Horwitz, 1997; Kristiansen and Mooney, 2004), focusing on the weaknesses of a generalised evidence base (Kirmayer, 2012), EBM's devaluation of professional judgement (Feinstein, 1994; Goldenberg, 2006) and patients' life narratives (Greenhalgh, 2014). Similar critiques have emerged from the social sciences, where authors have explored EBM cultural assumptions (De Vries and Lemmens, 2006), professional resistance toward EBM (Armstrong, 2007; Bhandari et al., 2003; Pope, 2003; Traynor, 2009) and its inability to remove professional judgement from clinical decision-making (Kelly and Moore, 2012; Mykhalovskiy, 2003; Mykhalovskiy and Weir, 2004; Upshur, 2005).

EBM practice has standardised the quality assessment of research evidence (Goldenberg, 2006; Lambert, 2006), thus creating hierarchies of evidence in medicine. In the EBM pyramid of evidence (for an overview see Murad et al., 2016), meta-analyses of randomised controlled trials (RCTs) and RCTs are ranked as top quality evidence, while non-randomised trials (such as cohort studies and case-control studies), qualitative, observational, and other small-scale studies are considered to be of low quality.

RCTs have had a central role in the diffusion of EBM (Devereaux and Yusuf, 2003). Including procedures such as blinding, randomisation, and placebo controls, the RCT design has gained dominance in research methodology for three main reasons: generalisability, causal inference and perceived non-bias. Although RCTs have significant and well‐known limitations (for a summary see Deaton and Cartwright, 2018), they have gained concrete authority as a method of investigation. Within medicine, a strict orthodox perspective has emerged where only good-quality RCTs and meta-analyses are seen as ‘hard evidence’ or the only acceptable way to establish the effectiveness of drugs and devices (Feinstein and Horwitz, 1997).

Recent debates on RCTs (see the special issue in this journal edited by Mowat et al., 2018) have seen reiterations of criticisms of the evidence hierarchy (Concato et al., 2000; Deaton and Cartwright, 2018). Methodological problems with trials (Timmermans and Berg, 2003) and meta-analyses (Moreira, 2007) as well as the everyday difficulties of producing experimental knowledge (Miles et al., 1997) have been thoroughly explored in the social science literature. Although the lack of RCTs does not always diminish confidence in therapeutic effectiveness (Borgerson, 2005), new treatments are often required to meet standards that many established medical practices have never actually met (Morreim, 2003).

Empirical studies of EBM enactment in medical practice have shown that evidence cannot replace professional knowledge and skills (Berg and Timmermans, 2000; Tonelli, 2006); rather, medical expertise and evidence are complementary forms of knowledge (Timmermans and Berg, 2003). Even when professionals do not resist EBM, they still rely on a variety of knowledge forms (Armstrong, 2002; Latimer et al., 2006; Rabeharisoa and Bourret, 2009). Similarly, a recent study (Hughes and Doheny, 2019) investigating Welsh NHS deliberations on high-cost drug funding, demonstrates how such debates must still consider organisational and lifeworld factors, not just ‘hard’ evidence.

## 3 The discourse of evidence in IVF

Over the years, IVF development has been technologically driven rather than evidence-based. Neither the early experimental procedures nor the following additional treatments (sperm injection being the prime example) have been routinely scrutinised through EBM protocols before their clinical introduction. An excessive enthusiasm for new technological interventions and their commercialisation has always been a source of concern in the professional community (Hurley, 2013). However, the current controversy on add-ons has arisen following BBC Panorama's request to Oxford University's Centre for Evidence-Based Medicine for an investigation. The resulting publications (Heneghan et al., 2016; Spencer et al., 2016) only included the ‘highest level of evidence’ in their analysis (e.g., Cochrane reviews and non-Cochrane systematic reviews), concluding that there is no data to suggest that any add-ons can improve LBRs and criticising the HFEA for not providing sufficient guidance to patients.

As a response, the HFEA started an independent assessment and in 2017 launched a webpage (HFEA, 2019b) where patients could find efficiency and safety information on the 9 most widely-used add-ons. This initiative aimed to improve informed choice ‘without completely denying patient-access to potentially beneficial innovations’ (Macklon et al., 2019). However, the HFEA also adopted a conventional EBM perspective on evidence, which is summarised in a traffic light system: green light, for treatments with more than one quality RCT; amber light, for treatments with a small or conflicting body of evidence and further research required; red light, where no evidence of safety or effectiveness exists. At the time of writing, none of the add-ons reviewed has a green light.

The controversy has generated heated debate in the medical literature, where professionals argued that, in IVF, ‘appropriately powered, well-designed, peer-reviewed RCTs, with an LBR outcome measure which go on to report on child health, are the gold standard of evidence based medicine’ (Harper et al., 2017, p. 489). Repeated pleas to conduct more quality RCTs before routinely offering treatments to patients proliferated in the professional community (Repping, 2019; Wilkinson et al., 2017). However, as some experts noted (Macklon et al., 2019), the pursuit of the gold standard has, so far, left professionals in a difficult position: lacking robust evidence to make clinical decisions, while being condemned for using unproven treatments.

A major obstacle emerging in evidence production is the criteria divide between EMB reviewers and IVF professionals (Dhont, 2013), with the former emphasising LBRs as the outcome, while professionals focus on pregnancy rates. A second problem is the number of participants required to demonstrate a clinically- and statistically-significant impact on LBRs (Stocking et al., 2019), where proving a 5% increase requires over 2500 patients (Wilkinson et al., 2019). In a few successful cases, when good quality RCTs were finally published, the treatments assessed were already obsolete (Macklon et al., 2019). Clinicians have attempted to circumvent the problem by using routinely collected data and large electronic databases. This, however, is not gold standard evidence due to the lack of random allocation. Finally, the lack of consistency in data reporting often precludes meta-analyses (Wilkinson et al., 2019).

TLI is a prime example of the problems related to evidence-production: while unanimously being considered safe by the professional community, no clear consensus exists over its ability to increase LBRs. Although several RCTs have been conducted, two Cochrane reviews (Armstrong et al., 2015, 2019) and several non-Cochrane meta-analyses concluded that available evidence is still insufficient to prefer TLI over standard incubators. Interestingly, reviewers also critiqued the low evidence quality (e.g., lack of data on LBRs, inconsistent reporting, poor blinding and randomisation procedures). Nonetheless, even orthodox EBM supporters suggest that TLI has further potential benefits that need to be explored in order to determine what its best role in the IVF laboratory is (Harper et al., 2017). Despite the heated debate on add-ons, the extended social science literature on IVF has overlooked how fertility professionals legitimise their use of treatments in clinical practice. This paper fills this gap by examining the legitimation narratives enacted by professionals and their understanding of evidence.

## 4 Narrative legitimation in medicine

The discursive legitimation of medical practices is an important tool that professionals employ in order to justify the use of novel treatments or technologies. In this article, we focus on a ‘narrative of legitimation’ analytical frame, which has been employed by other empirical studies of medicine (Baer, 2006; Foley and Faircloth, 2003; Lambert, 2012; Tausig and Subed, 1997). Authors have explored legitimation in naturopathy (Baer, 2006), midwifery (Foley and Faircloth, 2003) and local medical and healing knowledge (Lambert, 2012; Tausig and Subed, 1997).

Using the example of midwives' professional legitimation, Foley and Faircloth (2003, p. 168) argue that discourses are ‘not something conveyed in a narrative vacuum,’ but are rather ‘based on the interpretive wants and needs of the teller.’ As such, medical professionals will invoke a certain set of legitimation narratives (Foley and Faircloth, 2003). In the case of TLI, the three emerging dominant types of legitimation discourses employed by professionals are embedded in the current discourse of evidence in IVF.

The question of how professionals legitimise the use of alternative forms of evidence in their practice has been investigated extensively in the literature on complementary and alternative medicine (CAM) (Jackson and Scambler, 2007; Derkatch, 2016; Gibson, 2018). Although CAM treatments are found worldwide and are offered by many state care regimes, they have been ostracised by EBM supporters as ‘unproven medicine’ (Fontanarosa and Lundberg, 1998). Social studies of CAM have underlined the epistemological incompatibility between the orthodox EBM approach and holistic methods focusing on individualised care (Degele, 2005; Trnka and Stöckelová, 2019). Borgerson (2005) summarises three possible alternative options for professionals working towards legitimacy of CAM in the medical community: accept EBM standards and produce evidence accordingly; rely on lower evidence to support their decision; critically engage with EBM and propose alternative standards.

We argue in this article that IVF professionals share orthodox views on notions of acceptable evidence, while simultaneously offering alternative perspectives on the role of TLI in improving clinical practice.

## 5 Methodology

Data presented in this article are part of a larger project and were collected between June 2017 and March 2019. This included ethnographic observations and interviews carried out by both authors in NHS sites where fertility treatment is provided, including the use of TLI tools. Clinics were selected based on their frequent use of TLI, availability, and willingness to participate in the study. Clinics agreed to participate in the research study and all staff were informed ahead of time about study procedures. All clinics are located in England. Further details about location cannot be disclosed due to confidentiality concerns. In addition to university ethics approval, we received research clearance from the NHS and each clinic site separately.

Although our understanding of local IVF practices is supported by observations in the clinics, in this article, to explore how the use of TLI is legitimised by professionals, we focus on data from interviews only. IVF professionals working in the observation sites were personally approached by the researchers regarding interviews. Participation was voluntary and interviewees signed a consent form. We conducted a total of 25 interviews with NHS staff. A minority of interviewees were not lab staff, but had knowledge of TLI or had talked to patients about the use of such tools (for details, see Table 1). Interviews lasted between 45 and 90 min and were audio recorded and professionally transcribed. The interview guide was designed to obtain participants' experiences with using TLI, its challenges, benefits, and place in IVF treatment in the UK. The interviewers carefully elicited professionals’ views on the use of TLI as an add-on treatment and its evidence, without expressing any personal opinions on the matter.

**Table 1:** Data collection.

Semi-structured interviews	Heads of embryology	Senior embryologists	Embryologists	Clinical director	Fertility consultants	Research nurses	Nurses
25	4	5	9	1	2	2	2

Data analysis was carried out in two stages. In the initial stage of analysis, we entered all observation and interview data into NVivo, a software package for organising and analysing qualitative data. Following this, the data were coded both deductively, using themes derived from previous literature, and inductively, where we assessed new themes. A potential list of codes was agreed upon in advance. As new codes appeared, we cross-checked each other's work so as to reach consensus on the main emerging themes. Analysis of all data involved a continual process of literature review, coding, and memo writing. Grounded theory was primarily used to guide the process of analysis, from coding to developing larger themes (Glaser and Strauss, 2017). However, discourse analysis principles were also applied in order to take into account the social context in which the conversation occurred, including previous conversations and power relationships (Wodak and Meyer, 2015). We paid close attention to professionals': 1) understanding of the purpose of TLI; 2) opinion on the evidence available for TLI; 3) views on the production of evidence for IVF treatments; 4) views on technological development in IVF; 5) understanding of the fit of TLI in their clinic; 6) challenges experienced using TLI; and 7) experiences with patients who have had TLI included in their treatment.

The second stage of analysis focused on the interviews to examine the professional legitimation narratives. Using an interpretive practice and narrative legitimation approach (Gubrium and Holstein, 1997; Holstein and Gubrium, 2000), we paid attention to professionals' arguments in favour of the use of TLI in fertility clinics. Interpretive practice can be conceptualised as the way in which respondents construct reality through storytelling (Holstein and Gubrium, 2000). Interpretive practice, however, is also related to respondents' context and the experiences they draw upon to explain their practices. In the case of TLI, our interviewees drew upon their experience with this technology and their perception of patients’ needs and wants. The narrative legitimation approach has allowed us to analyse the data without making a value judgement on the appropriateness of TLI and its use. It has also allowed us to highlight professional narratives and how they legitimise the use of a technology in relation to the dominant discourse of EBM.

One of the limitations of our sample is that clinics where TLI is integrated into daily practice were more likely to participate in the research. We are aware that there are clinics where staff are more resistant to incorporating such technologies into practice. Our access to participants from such clinics was limited. Out of the 5 clinics where the research took place, 4 put all or most patient embryos in TLI incubators. This practice is different from some private clinics where patients pay an extra fee for the inclusion of TLI in their treatment. However, some of the professionals interviewed had previous experience of working in private clinics and talked about this during their interviews. Nonetheless, our data can only speak for NHS/semi-public clinics where efforts have been made to incorporate TLI into daily lab practice. That being said, our understanding is that the integration of TLI technologies into UK clinics, and particularly NHS ones, is rapidly expanding.

## 6 Findings

Three main legitimation narratives emerged in our analysis: the lab usefulness of TLI, its potential for knowledge creation, and its ability to support patient needs. The strategies that professionals use to legitimise the use of TLI tools in the lab emphasise technology benefits that are not necessarily captured by EBM discourses. Nonetheless, these strategies were not a direct critique of the value placed on RCTs by modern medicine. We found that interviewees did not question the EBM paradigm. Rather, they agreed that more high-quality evidence is needed, but were generally hopeful that TLI will prove to be beneficial in clinical practice.

### 6.1 Time-lapse as lab tool

TLI is quite different from other add-ons offered in the UK in that it does not involve any invasive bodily procedures. For example, the endometrial scratch disrupts a woman's endometrium prior to embryo transfer, while intralipids are administered to the patient intravenously. Due to their invasive nature, such procedures carry more negative connotations. TLI technologies, on the other hand, are not meant to treat infertility, but rather help in the selection of a transfer embryo. Interestingly, we found that professionals often categorise TLI tools as lab equipment. This distinctive feature of TLI played an important role in interviewees' understanding of it, its purpose, and the need for evidence. Most notably, they stressed that the tool has been a positive change for the lab and its routine. This was seen as an advantage that could compensate for the lack of conclusive efficiency data. Two embryologists explained the significance of TLI as a lab tool:

*But time-lapse has so many other uses, right, for research and knowledge and understanding everything. And it is convenient and embryologists don't have to come at 7.30 in the morning and check for fertilisation because you won't miss fertilisation, that is one of the other things. Because traditionally, without time-lapse, what we do, we put the sperm and the eggs together or inject, and the pro-nucleus appears and then it disappears, so we don't know when it appears but we want to catch it before it disappears […] But with time-lapse there is no problem. So you can go back and see from first hour to the 20th hour. (Head of embryology, Clinic D).*

*Time lapse is a modern way of working. It's like, you know, I'm quite tech focused so I like going in and being able to look at embryos and see what they did […] for me I just like when embryos go into time-lapse so you get to look at them. (Embryologist, Clinic A).*

TLI has helped embryologists monitor embryos in a convenient and efficient way. It has removed the need to take embryos out of the incubator once a day to assess them under the microscope. We observed that it has also provided comfort to staff that important events such as nucleation and fertilisation are not missed.

By emphasising its merits as a lab tool, professionals tried to counter the dominant narrative that TLI is only of value if it can improve LBRs. Many seemed hopeful, however, that the evidence will come out eventually and it is just a matter of time before TLI proves its worth beyond the lab. For most, the routine monitoring benefits were salient in their evaluation of this new technology. Professional perception of TLI contrasts with the initial marketing campaigns for such tools – campaigns that promised dramatic increases in pregnancy rates for patients. We observed a significant disconnect between the initial promissory discourses surrounding TLI as ‘treatment’ and the practical purpose that it serves in IVF labs currently. Professional views showed ambiguity regarding the purpose of TLI. Consequently the role of evidence also became unclear due to EBM's inability to fully capture the purpose and multiple uses of TLI.

The ability to practice EBM was also contrasted with the rapid pace of innovation in the field. Interviewees expressed frustration with the hope and hype brought about by the launch of new tools and treatments in IVF. On one hand, they acknowledged the importance of evidence, but on the other hand, they felt technology is moving too fast in this ever-changing field of medicine. This conundrum was summed up by one respondent:

*Things are developing so quickly that trying to get a true randomised control trial seems that you're just putting the clock back because things are already moving further and further down the line. So we have to deal in some cases where there is evidence by smaller studies, by whatever else and have an idea and that will at least, pilot studies will at least put you in the position to say right, this is more interesting and at least you can exclude the stuff that doesn't work at that point. (Clinical director, Clinic B).*

Due to the fast pace of technological change in fertility care over the past few decades, the task of conducting RCTs and waiting for results was seen as unfeasible by many interviewees. Professionals did value evidence, but they felt that there are bigger commercial and organisational forces that dictate the treatments that their clinics decide to offer. They talked about having to play ‘catch up’ with private clinics who adopt new technologies more hastily. Professionals felt that the commercial logic of fertility medicine in the UK does not leave much room for regulation informed by evidence. Nevertheless, TLI is a technology that interviewees felt positively about, especially in light of its practical uses in the lab.

The conceptualisation and legitimation of TLI as a lab technology lent itself to another advantage: the knowledge that this add-on does no harm to the patient or their embryos. Consequently, the need for evidence seemed slightly less urgent than in the case of invasive procedures. Professionals were not naïve regarding the yet unproven status of TLI as an efficient add-on. However, they stressed that, in theory, uninterrupted culture must be better for embryos as they are not exposed to potentially damaging outside conditions. This, in turn, downplayed the immediate need for evidence. For example, one interviewee elaborated on the relationship between harm and evidence:

*Even if you tell them [patients] that there's no evidence, they may think okay, there is no evidence that it helps but there is definite evidence that it doesn't do any harm. In which case they go for that aspect. If you tell them that putting embryos in time lapse will harm the embryos they will definitely say I don't want to do it. But if you tell them that we don't know whether it helps or not, you may try it if you want to, and then they'll say okay, fine, there is no harm at least. (Fertility consultant#1, Clinic D).*

When the possibility to practice EBM was constrained, professionals looked for alternative ways to judge a new technology and decide whether or not it should be offered to patients. In the case of TLI, some of its intrinsic features, such as its uses as a lab tool and very low harm potential, worked to its advantage and timely adoption.

### 6.2 Potential for knowledge production

Evaluating TLI only in terms of efficiency would not capture its potential for knowledge production, according to many of our interviewees. Professionals highlighted the complexities of embryo development and the importance of additional information made available to embryologists by TLI technologies. A salient benefit was the ability to deselect embryos that behave abnormally. Abnormal events would be harder to observe without TLI because the embryos would not be monitored 24/7. An embryologist explains:

*I'd still preferentially use it because I think there is enough evidence to say like we said before about the direct cleavage embryos and you've got a much better time frame to see fertilisation results. We sometimes see, you know, we've put an embryo in and called it normally fertilised, it's got two PNs [pronuclei], and then a couple of hours later a third PN pops up and you'd never in a static observation, we never would have picked that up and we could have possibly transferred that embryo knowing that as a three PN we would know that it’s unlikely, we know it's abnormal so we wouldn't want to implant, and are unlikely to implant it, wouldn't want it to because it's got too much DNA, it's abnormal. […] So although it might not be saying this is your one embryo that needs to go back, it's telling us which embryos to definitely not put back. So I think on the back of the fact that it’s a good incubator and that is a benefit to some people I would, I'd still preferentially use the time lapse over a benchtop, yeah. (Head of embryology, Clinic E).*

Here, the embryologist emphasises abnormal embryo development events, such as direct cleavage where an embryo goes from 2 to 3 cells too quickly (<5 h) or the presence of three pronuclei (PN) instead of the normal two. TLI has allowed professionals to deselect the embryos which are of lower quality based on the observation of such events that would normally be missed with standard incubation. Correlations between embryo behaviour and patient outcome are just now starting to get research attention in the field, due to the availability of TLI.

TLI technologies, no doubt, offer embryologists more information about embryos than was ever available. From a professional point of view, it is difficult to argue that additional knowledge is not needed, especially in a fast-changing field such as IVF. We observed that being able to deselect embryos based on detected abnormal events was a benefit that lab professionals stressed repeatedly. Conscious, however, that this has not necessarily translated into RCT evidence of effectiveness, interviewees talked about the unknown and underexplored potential of TLI. For instance, TLI could reduce time to live birth by optimising the embryo selection process. One respondent, for example, legitimised the use of TLI by highlighting the need for collective efforts to develop knowledge in embryology:

*Although there aren't these gold standard multi-centred randomised control trials that show that time lapse is the best, it's a bit logical really that you have an incubator that works as an incubator but also it can capture images of embryos. It does no harm, we know it does no harm but, it's because we don't know what to do with the information yet that people are a bit, still a bit, there's camps, isn't there, people that don't think that it’s worthwhile and it's very expensive. But I think that's down to the lack of knowledge about what to do with the information because we don't, we're getting all this information, what do we do with it, we just need to work that out. (Head of embryology, Clinic B).*

Professionals stressed that there might be extra steps required to prove that TLI is worthwhile, but were hopeful that more information can only be a positive aspect for both lab staff and patients. They also talked about the benefit of knowledge production in order to counter the evidence discourse. RCTs were often seen as lengthy and unfeasible. Many professionals felt it would take an unbearably long time to produce evidence of efficiency. This is, of course, not to say that the intrinsic value of RCTs was not talked about. Most respondents did wish to see more RCTs conducted on new IVF add-on treatments. However, they had more nuanced ways to evaluate and legitimise the use of new technologies, including the aforementioned benefits that they highlighted in the case of TLI. Given the low success rates and many unknowns in IVF treatment, TLI was described by respondents as a knowledge production tool with great potential – even if that potential is not fully apparent yet. While in its early days, TLI has been problematically portrayed in some marketing campaigns as an entirely AI-powered selection tool, our research has found that it is rather seen as a tool that gives professionals confidence in deselecting abnormal embryos, thus narrowing the transfer pool. TLI also promises more knowledge about embryos and their behaviour in the near future.

### 6.3 Managing patient hopes and expectations

The public conversation on add-ons and evidence has brought concerns about commercialisation and patient vulnerability. The main issue highlighted by the HFEA and other professional organisations is that of fertility patients paying exorbitant sums of money for unproven treatments. Although TLI is categorised as amber by the HFEA, meaning that it shows some promise, there is currently no definitive evidence that it improves LBRs. While professionals were indeed concerned about commercialisation, they also talked about the dilemma of balancing patient hopes and expectations with institutional approaches to evidence. Two respondents elaborated on the pressure to help patients right now, while also trying to wait for more evidence to become available:

*The trouble with IVF is that there isn't many RCTs because no one wants to go in an RCT, they [patients] want that improvement now. Patients don't want to wait. They don't want to be a guinea pig in a trial, they just want it now. So clinics are under pressure to introduce it straightaway, they're under pressure to improve success rates, and I think that's fine if you're not charging for it. (Embryologist, Clinic C).*

*Then some people might also say that when is this super quality RCT going to come? Who is going to pay for it? It might take ten years before this evidence comes out. So until then what are we supposed to do because the patient wants to get pregnant now. (Fertility consultant#2, Clinic D).*

There was a tension between the care responsibility that professionals felt towards patients and the public disapproval of add-ons. As the respondents above mention, ‘patients don't want to wait,’ ‘they want to get pregnant now’ and professionals want to do everything possible to help them have a baby. The discourse of patient needs served as further legitimation for the use of new treatments.

Respondents also understood that they need to manage patients' expectations and deal with the promissory discourses that often surround new technologies in IVF. This means that sometimes they might offer emerging treatments, provided that risks and benefits are discussed thoroughly. As an embryologist from Clinic B put it, ‘*if a patient wants something and you can offer it to them and you counsel them for all of the benefits and risks, then why wouldn't you give it to them.’* Some interviewees talked about fertility care being very ‘patient driven’ where patients might demand certain treatments they have researched online or have heard of in the media. Consequently, NHS professionals struggled with the idea of denying patients access to some treatments if they could access them in a private clinic instead. Professionals who worked in clinics offering TLI as part of their standard package and not as an add-on felt that the technology should be offered routinely. However, they also stressed that it is acceptable to offer TLI as an add-on as long as patients are aware of the lack of evidence and make informed choices.

Giving patients the ability to make informed decisions was seen by interviewees as the best way to guide treatment choices in light of the limited evidence available. Fertility consultants (usually gynaecologists specialised in reproductive medicine) and embryologists also valued the ability to give patients more in-depth information obtained through TLI. They emphasised that such technologies can build pathways towards increasingly personalised treatment, as the following embryologist explained:

*So we're a little bit more transparent and for patients that might help them make a decision whether to have another cycle or if they have that information and they're like could it be my eggs, could it be the sperm, shall I use donor eggs next time, shall I use donor sperm. […] so it allows us to give patients more information and kind of if embryos have done kind of unusual things up until day three, we can kind of warn patients that we might need to cancel the transfer on day five so it's, we use it to kind of manage patient expectations as well. So I quite like it for that. I don't ever want to go back. (Senior embryologist, Clinic E).*

The interviewee stresses that TLI is beneficial for giving patients more information about the source of their infertility, especially if they struggle to get pregnant. Professionals are acutely aware that patients want answers and see TLI as playing a role in deciding the best course of treatment.

Overall, professionals felt very much constrained by the commercialisation of the UK fertility sector. Their views on the use of EBM guidelines in IVF reflect the ambiguous role that evidence currently plays in their field. On one hand, institutional stakeholders decry the lack of quality RCTs to prove treatment effectiveness. On the other hand, care professionals have to juggle expectations and a commercially driven innovation logic where technologies are often adopted before enough evidence is available. TLI especially illustrates the difficulties they might face when evaluating new technologies and deciding whether or not to offer them to patients. As we have stressed here, evidence is not the only concern that drives clinical practice in IVF. As a result, we suggest, in the discussion section, a more nuanced approach to thinking about the role of evidence in fertility treatment.

## 7 Discussion

Despite the heated debate on the availability of evidence in support of add-ons, the extended social science literature on IVF has overlooked how professionals legitimise their current use of additional treatment in their clinical practice. This paper helps fill this gap by examining the legitimation narratives enacted by professionals and by focusing on their understanding of evidence. Our findings show how individuals deal with ambiguity when robust and definitive evidence is not available to support their clinical decision making. We extend previous findings that healthcare staff have to rely on diverse forms of evidence (Armstrong, 2002; Latimer et al., 2006; Rabeharisoa and Bourret, 2009; Tonelli, 2006). Interestingly, in the case of fertility care, professionals do not dispute orthodox EBM and do demand good quality RCTs to prove the advantages of TLI. However, they simultaneously downplay the role of hard evidence in understanding the benefits of TLI. As we have shown, their negotiation of legitimacy focuses on three main narratives: presenting TLI as an innovative piece of laboratory equipment; highlighting its crucial role in the production of knowledge; and the ability of these tools to offer additional information to manage patient hopes and expectations.

Contrary to what emerged in studies of CAM, fertility professionals do not engage with one of the three possible alternative strategies identified (Borgerson, 2005), but seem to produce more articulated legitimation narratives that include them all. They cosmetically accept EBM standards and call for more RCTs, whilst relying on professional experience and lower evidence to legitimise their use of TLI. At the same time, they critically engage with EBM, downplaying the role of evidence in the process of evaluating the worth of the tool. Although there are some calls for a more inclusive approach to evidence (such as in the case of CAM, see Trnka and Stöckelová, 2019), fertility professionals do not ultimately challenge the pyramid of evidence or the gold standard of RCTs. Instead, they articulate their critiques of evidence production in a more subtle way, using their professional expertise to challenge the application of a pure EBM discourse to fertility treatments.

Firstly, the emerging legitimation narratives question the prime purpose of TLI, redefining its role in supporting clinical practice as a sophisticated piece of lab equipment, rather than an add-on that increases LBRs. Broadening the acceptable interpretation of what TLI is and what it does allows them to focus on its multiple benefits and its technical advantages (such as monitoring benefits, uninterrupted culture, data production). By refocusing their attention on the benefits of TLI as support for laboratory work and tool for knowledge production and patient support, fertility professionals effectively devalue the role of hard evidence. This is due to EBM's inability to include multiple perspectives on treatment benefits (for instance, the great support offered by TLI to ‘deselect’ poor quality embryos and therefore reduce the time to live birth). Thus, the case of TLI use in IVF further illustrates the importance of care standards and the role professionals themselves play in clinical decision-making (Kelly and Moore, 2012; Mykhalovskiy, 2003; Mykhalovskiy and Weir, 2004; Upshur, 2005). Professionals also stress the tool's potential to develop the field of embryology itself, in light of TLI's ability to provide a large amount of embryo development data. Such information was not available in the past and can offer future opportunities.

Secondly, through their legitimation narratives, fertility professionals redefine the notion of effectiveness (LBRs) as adopted by orthodox EBM. By stressing TLI benefits as a lab tool and emphasising its potential, professionals challenge the dominant narrative that TLI is only of value if it can improve LBRs. They focus instead on a more holistic interpretation of potential benefits, where the ability of TLI to provide more information for professionals and patients is highly valued. Interestingly, the interpretation of LBRs as the only criterion of success is shared across a variety of actors in the field as a response to very different logics and interests. From an EBM and regulatory perspective, LBRs are the only outcome measure that allows objective clinical performance comparisons. For patients and clinics, however, success rates (expressed in pregnancy or delivery rates) are the easier way to compare clinic performance. The stress on LBRs as the only performance indicator has been highly criticised in the field (HFEA, 2019c) due to the measure's neglect of other relevant factors, including the variation in patient populations. In addition, a focus on LBRs does not account for clinical and individual decisions that influence the outcome but cannot be made on the basis of clinical performance only. For example, single embryo transfers are a safer option to avoid multiple pregnancies and related health risks, but reduce success rates. Similarly, using donor eggs to treat older patients can dramatically increase success rates, but has individual and moral implications. Therefore, the focus with TLI on multiple uses and benefits is typical in a field characterised by high uncertainty and moral and ethical issues. As shown by other studies (Berg and Timmermans, 2000), we stress that evidence cannot replace professional knowledge. Professional judgment of TLI's value relies on a variety of knowledge forms (Armstrong, 2002), including forms of medical expertise not based on EBM (Timmermans and Berg, 2003).

Thirdly, our findings show how professional legitimation narratives challenge the emerging notion of ‘add-on’ treatment itself (Heneghan et al., 2016; HFEA, 2019b; Spencer et al., 2016). The current discourses, focusing on the lack of effectiveness evidence for additional treatments, have created a novel homogeneous category based on two main criteria: their being an addition to the ‘standard’ IVF treatment; and their cost. Focusing on laboratory work and practices, professionals offer a situated understanding of TLI and differentiate it from other add-ons on the basis of its varied advantages for clinical practice. Interviewees' legitimation narratives imply a further critique of the current add-on discourse. Instead of asking if it is ethical to offer a treatment at an additional cost where there is no evidence of LBR increases, professionals questioned whether it would actually be unethical not to offer an available tool with potential (provided that patients are informed of evidence and risks). Nonetheless, respondents acknowledge the ethical dilemma of offering treatments to vulnerable patients, positioning it in the broader context of a highly privatised sector.

## 8 Conclusion

To conclude, our findings show how fertility professionals are enacting, through the use of nuanced legitimation narratives, a novel form of resistance to the orthodox EBM discourse that does not openly challenge its rules or the concept of ‘hard evidence’. Rather, professional legitimation narratives question the premises of EBM and challenge the relevance of evidence, in a field still characterised by a high level of uncertainty and very limited knowledge on the reproductive process. The ability of TLI tools to answer previously inaccessible questions without causing any harm is central to the optimistic enthusiasm for them. Although the case investigated is central in the current debate on add-ons, due to the situated and specific nature of TLI, further research is needed to explore whether similar dynamics emerge in legitimation narratives of other treatments and devices. Another limitation of this research is that it is not able to speak for professionals working in private clinics where commercialisation plays an even more important role in the choice of treatments offered to patients. Nonetheless, our data demonstrates how the enactment of professional legitimation narratives takes place in the public sector in the UK. The way care professionals in the NHS conceptualise new treatments can affect future policy directions and inequalities in accessing IVF treatments across the UK.

The case of TLI illustrates larger issues within IVF. Clinicians feel pressure to improve outcomes immediately, given the commercial nature of the industry. In addition, regulation in the UK focuses on EBM to the extent where new technologies are not fully understood for all the benefits they can offer. Such forces put NHS professionals in a difficult position, especially given the lack of coordination and funding for RCTs. In light of the data presented here, we suggest that, as an emerging field, fertility care needs a more nuanced understanding of the relationship between innovation and evidence.

## Credit author statement

**Manuela Perrotta:** Conceptualization, Methodology, Investigation, Writing - Original Draft, Writing - Review & Editing, Supervision, Project administration, Funding acquisition. **Alina Geampana:** Conceptualization, Methodology, Investigation, Formal analysis, Data Curation, Writing - Original Draft, Writing - Review & Editing.

## Figures and Tables

**Table:** 

Document	Version	Date
Confirmation of any other Regulatory Approvals (e.g. NIGB) and all correspondence [Name of University Ethics approval for stage I]		30 November 2016
Confirmation of any other Regulatory Approvals (e.g. NIGB) and all correspondence [Name of University Ethics approval for stage II]		30 March 2017
Copies of advertisement materials for research participants [Call for participants]	1	06 April 2017
Covering letter on headed paper		19 May 2017
Evidence of Sponsor insurance or indemnity (non NHS Sponsors only)		15 July 2016
Interview schedules or topic guides for participants [Professionals]	1	20 March 2017
Interview schedules or topic guides for participants [Patients]	1	20 March 2017
Interview schedules or topic guides for participants [Focus group guide]	1	20 March 2017
IRAS Application Form [IRAS_Form]		24 May 2017
Laboratory Manual [Schedule of events]		09 May 2017
Letter from funder [Funding body - Acceptance letter]		24 September 2015
Letter from sponsor [Provisional Sponsorship letter]		19 May 2017
Other [Schedule of Events]	1	30 May 2017
Other [Statement of Activities]	1	30 May 2017
Other [CI Research Governance training]		04 April 2017
Other [Postdoc Research Governance training]		04 April 2017
Participant consent form [Interviews - patients]	1	09 May 2017
Participant consent form [Focus groups - professionals]	1	09 May 2017
Participant consent form [Focus groups - patients]	1	09 May 2017
Participant consent form [Sharing information]	1	09 May 2017
Participant consent form [Observations - professionals]	1	09 May 2017
Participant consent form [Observations - patients]	1	09 May 2017
Participant consent form [Interviews - professionals]	1	09 May 2017
Participant information sheet (PIS) [Observations - professionals]	1	09 May 2017
Participant information sheet (PIS) [Observations - patients ]	1	09 May 2017
Participant information sheet (PIS) [Interviews - professionals]	1	09 May 2017
Participant information sheet (PIS) [Interviews - patients]	1	09 May 2017
Participant information sheet (PIS) [Focus groups - professionals]	1	09 May 2017
Participant information sheet (PIS) [Focus groups - patients]	1	09 May 2017
Referee's report or other scientific critique report [Peer reviewers' comments on full project]		10 June 2015
Referee's report or other scientific critique report [Department support letter]		30 March 2015
Research protocol or project proposal [Research Protocol]	1	09 May 2017
Summary CV for Chief Investigator (CI) [PI - Short CV]	1	06 April 2017
Summary CV for student [Postdoc CV]		06 April 2017
Summary, synopsis or diagram (flowchart) of protocol in non technical language	1	09 May 2017

**Table:** 

Section	HRA Assessment Criteria	Compliant with Standards	Comments
1.1	IRAS application completed correctly	Yes	No comments

2.1	Participant information/consent documents and consent process	Yes	No comments

3.1	Protocol assessment	Yes	No comments

4.1	Allocation of responsibilities and rights are agreed and documented	Yes	A Statement of Activities and Schedule of Events have been provided for use with participating NHS organisations in England. Exchange of the Statement of Activities will act as confirmation of capacity and capability for an organisation to host the research.
4.2	Insurance/indemnity arrangements assessed	Yes	Insurance for the management and design of the study will be provided by the sponsor, and indemnity for the conduct by the NHS. Where applicable, independent contractors (e.g. General Practitioners) should ensure that the professional indemnity provided by their medical defence organisation covers the activities expected of them for this research study
4.3	Financial arrangements assessed	Yes	External study funding has been secured from the Funding Body. Funding is not available to participating NHS organisations.

5.1	Compliance with the Data Protection Act and data security issues assessed	Yes	No comments
5.2	CTIMPS Arrangements for compliance with the Clinical Trials Regulations assessed	Not Applicable	No comments
5.3	Compliance with any applicable laws or regulations	Yes	No comments

6.1	NHS Research Ethics Committee favourable opinion received for applicable studies	Yes	The study has a favourable ethical opinion from the XXX Research Ethics Committee.
6.2	CTIMPS Clinical Trials Authorisation (CTA) letter received	Not Applicable	No comments
6.3	Devices MHRA notice of no objection received	Not Applicable	No comments
6.4	Other regulatory approvals and authorisations received	Not Applicable	No comments

## Appendix A List of Documents

The final document set assessed and approved by HRA Approval is listed below.

## Appendix B Summary of HRA Assessment

This appendix provides assurance to you, the sponsor and the NHS in England that the study, as reviewed for HRA Approval, is compliant with relevant standards. It also provides information and clarification, where appropriate, to participating NHS organisations in England to assist in assessing and arranging capacity and capability.

For information on how the sponsor should be working with participating NHS organisations in England, please refer to the, participating NHS organisations, capacity and capability and Allocation of responsibilities and rights are agreed and documented (4.1 of HRA assessment criteria) sections in this appendix.

The following person is the sponsor contact for the purpose of addressing participating organisation questions relating to the study:

Name: Author One
Tel:
Email:

HRA assessment criteria
